# The use of quantitative T1-mapping to identify cells and collagen fibers in rectal cancer

**DOI:** 10.3389/fonc.2023.1189334

**Published:** 2023-07-20

**Authors:** Jie Yuan, Qun Wen, Hui Wang, Jiaoyan Wang, Kun Liu, Songhua Zhan, Mengxiao Liu, Zhigang Gong, WenLi Tan

**Affiliations:** ^1^ Department of Radiology, Shuguang Hospital Affiliated to Shanghai University of Traditional Chinese Medicine, Shanghai, China; ^2^ Department of Pathology, Shuguang Hospital Affiliated to Shanghai University of Traditional Chinese Medicine, Shanghai, China; ^3^ MR Scientific Marketing, Diagnostic Imaging, Siemens Healthineers Ltd, Shanghai, China

**Keywords:** magnetic resonance imaging, T1 mapping, extracellular volume, rectal cancer, tumor tissue composition

## Abstract

**Aim:**

This study aimed to explore the value of T1 mapping in assessing the grade and stage of rectal adenocarcinoma and its correlation with tumor tissue composition.

**Methods:**

Informed consent was obtained from all rectal cancer patients after approval by the institutional review board. Twenty-four patients (14 women and 10 men; mean age, 64.46 years; range, 35 – 82 years) were enrolled in this prospective study. MRI examinations were performed using 3.0T MR scanner before surgery. HE, immunohistochemical, and masson trichrome-staining was performed on the surgically resected tumors to assess the degree of differentiation, stage, and invasion. Two radiologists independently analyzed native T1 and postcontrast T1 for each lesion, and calculated the extracellular volume (ECV) was calculated from T1 values. Intraclass correlation coefficient (ICC) and Bland-Altman plots were applied to analyze the interobserver agreement of native T1 values and postcontrast T1 values. Student’s t-test and one-way analysis of variance (ANOVA) were used to test the differences between T1 mapping parameters and differentiation types, T and N stages, and venous and neural invasion. Pearson correlation coefficients were used to analyze the correlation of T1 mapping extraction parameters with caudal type homeobox 2 (CDX-2), Ki-67 index, and collagen expression.

**Results:**

Both the native and postcontrast T1 values had an excellent interobserver agreement (ICC 0.945 and 0.942, respectively). Postcontrast T1 values indicated significant differences in venous invasion (t=2.497, *p*=0.021) and neural invasion (t=2.254, *p*=0.034). Pearson’s correlation analysis showed a significant positive correlation between native T1 values and Ki-67 (r=-0.407, *p*=0.049). There was a significant positive correlation between ECV and collagen expression (r=0.811, *p*=.000) and a significant negative correlation between ECV and CDX-2 (r=-0.465, *p*=0.022) and Ki-67 (r=-0.549, *p*=0.005).

**Conclusion:**

Postcontrast T1 value can be used to assess venous and neural invasion in rectal cancer. ECV measurements based on T1 mapping can be used to identify cells and collagen fibers in rectal cancer.

## Introduction

Cancer is a significant contributor to the global disease burden. Colorectal cancer is one of the leading causes of cancer-related deaths worldwide, accounting for approximately 9.4% of all malignant tumors (1). Accurate diagnosis and evaluation of colorectal cancer are essential for individualized treatment planning and prognosis assessment.

Magnetic resonance imaging (MRI) is routinely used to evaluate rectal cancer because of its excellent soft tissue resolution and multiparametric imaging without ionizing radiation. T1 mapping is a high-resolution longitudinal relaxation time imaging technique with high sensitivity and tissue specificity that can quantify directly to native T1 values. This reflects subtle differences in composition and water content within tissues, quantifying *in vivo* histology and determining biological tissue characteristics (2).

Postcontrast T1 mapping refers to the longitudinal relaxation time images after the contrast agent injection. It can be used to differentiate radio necrosis and tumor recurrence, and it leads to a shortening of the tissue relaxation time T1 when a contrast agent accumulates in tumor tissue outside the vessels (3). The native and postcontrast T1 values after injection of the gadolinium contrast agent can be used to calculate the extracellular volume (ECV). This is a quantitative parameter that represents the percentage of non-cellular tissue space. In recent years, ECV scores have been used as valuable tumor imaging markers to predict tumor grade, response after chemotherapy, and overall survival of cancer patients (4–6).

Tumor composition and characteristics can be used to assess physiological and pathological processes or pharmacological responses to therapeutic interventions. A previous study found that the apparent diffusion coefficient (ADC) derived from diffusion-weighted imaging (DWI) had a significant inverse correlation with Ki-67 (7). Multi-parametric breast MRI correlated with the stroma component of invasive breast cancer (8).

However, the clinical value of T1 values and ECV based on T1 mapping in tumor evaluation and component recognition of rectal cancer remains unclear. Our study aimed to evaluate the potential of the T1 mapping technique in the evaluation of rectal cancer and to identify the components of rectal cancer.

## Methods

### Participants

**Figure 1:**
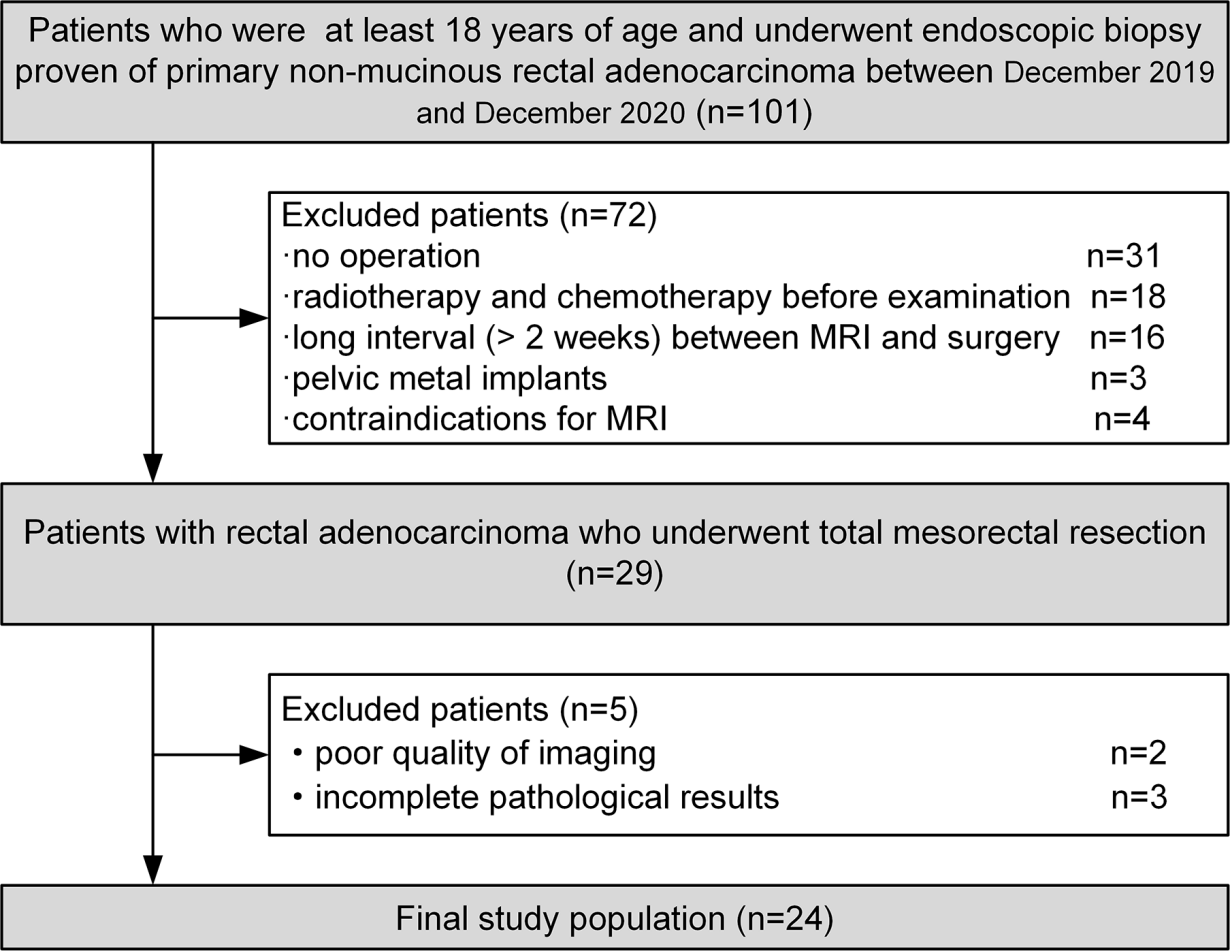
Flow diagram of patient selection for this study.

The institutional research ethics committee approved the prospective study (2019–750–105–01). Informed consent was obtained from all patients before participation. The inclusion criteria were: at least 18 years of age and primary non-mucinous rectal adenocarcinoma diagnosed by endoscopic biopsy. Between December 2019 and December 2020, 101 consecutive patients were enrolled in this study. The exclusion criteria were as follows: radiotherapy or chemotherapy before the examination, long interval (> 2 weeks) between MRI and surgery, contraindications for MRI, and poor imaging quality. Clinical and biochemical data were collected, including gender, age, and hematocrit levels, within 48 hours prior MR examination. **Figure 1** illustrates the patient selection process. This prospective observational study included 24 participants (14 women and 10 men) with rectal cancer. The mean patient age was 64.46 ± 12.55 years (range 35-82 years). The patient characteristics for this study are summarized in **Table 1**.

**Table 1:** Characteristics of 24 Patients with rectal cancer selected for this study.

Characteristic	Value
Patient sex	
No. of male	10(42%)
No. of female	14(58%)
Age (y)	
All patients	64.46 ± 12.55 [35-82]
Male	61.70 ± 10.14 [41-76]
Female	66.43 ± 14.05 [35-82]
*P*	0.375
T stages	
pT2	3(12.5%)
pT3	21(87.5%)
pT3a	11(45.8%)
pT3b	4(16.7%)
pT3c	6(25.0%)
N stages	
pN0	12(50.0%)
pN1	6(25.0%)
pN2	6(25.0%)
Venous invasion	
Negative	15(62.5%)
Positive	9(37.5%)
Nerve invasion	
Negative	16(66.7%)
Positive	8(33.3%)
Histologic Grades	
Low Grade	6(25.0%)
Medium Grade	12(50.0%)
High Grade	6(25.0%)

### MRI acquisition

Patients with histologically confirmed rectal adenocarcinoma underwent preoperative multiparametric MR imaging with a 3 Tesla MR unit (MAGNETOM Skyra, Siemens Healthcare) with an externally dedicated 18-channel body coil. All patients underwent routine clinical rectal imaging and received native and postcontrast T1 mapping.

Axial T1 mapping sequences were acquired using variable flip angle T1-weighted VIBE sequences with dual flip angles (2° and 15°). T1 mapping included imaging before and approximately 5 minutes after Gd-DTPA intravenous administration. Postcontrast T1 mapping was performed after 0.2 mmol/kg gadolinium-diethylenetriamine pentaacetate (Gd-DTPA) was administered intravenously using high-pressure syringes at 2 mL/s. The sequence was performed with the following parameters: TE, 1.47 ms; TR, 4.09 ms; acquisition matrix, 192 × 154; field-of-view, 200 × 200 mm; slice thickness, 2 mm; voxel size, 1.0 × 1.0 × 2.0 mm; 80% phase resolution, 6/8ths partial Fourier, and GRAPPA rate 2; bandwidth, 390 Hz/pixel. The acquisition time of the T1 mapping sequence was 3 minutes and 16 seconds.

### T1 mapping analysis

All images were independently examined by a radiologist with 8 years of experience and a radiologist with 11 years in rectal MR imaging. The radiologists are blinded to the patient’s information and clinical data. After comparison with T2WI, regions of interest (ROIs) were selected for delineation in the most profound plane of cancer infiltration. ROIs should be at least 1 cm^2^, avoiding bleeding, necrotic cystic areas, intestinal contents, and the mesentery. For each ROI, the mean native T1 and post-contrast T1 were recorded and used in the final analysis. The extracellular volume (ECV) fraction of rectal cancer was calculated using the equation below (9):


$$ECV=\left(1-\text{haematocrit}\right)\times \frac{1/\text{T}1\;\left(\text{post contrast T}1\text{ tumor}\right)-1/\text{T}1\;\left(\text{native T}1\text{ tumor}\right)}{1/\text{T}1\;\left(\text{post contrast T}1\text{ blood}\right)-1/\text{T}1\;\left(\text{native T}1\text{ blood}\right)}$$

### Surgical resection and histopathological staining

All surgical resections of total mesorectal excision were performed by the same surgeon team, with 20 years of experience in gastrointestinal surgery. After surgery, freshly excised rectal specimens were fixed in formalin for at least 24 h. After formalin fixation, the specimens were processed using a standard histological procedure before sectioning in a plane perpendicular to the long axis of the intestine. A pathologist with 18 years of experience and a radiologist with 8 years of experience selected the deepest rectal cancer infiltrations for sectioning based on T2W images. The sections were then processed and stained with hematoxylin-eosin (HE). A standard immunohistochemistry (IHC) protocol was used to stain rectal cancer tissues using caudal type homeobox transcription factor 2 (CDX-2) antibody (RayBiotech Cat# ER-14-0302, RRID: AB_1544774), and Ki-67 antibody (Spring Bioscience Cat# M3060, RRID: AB_1661313). A ready-to-use kit (Sigma-Aldrich) was used to identify collagen fibers for masson trichrome staining. CDX-2 staining was performed to determine the nuclei of the epithelial cells. Ki-67 staining was used as a proliferation marker for cancer cells.

### Histopathological evaluation

Whole-mount immunohistochemistry (CDX-2 and Ki-67) and masson trichrome-stained sections were generated and digitized at histological resolution. All the slides were scanned using a MoticEasyScan scanner at 20x magnification. We analyzed the tumor’s expression areas of CDX-2, Ki-67 (brown color), and masson trichrome staining (blue color). The IHC profiler plugin with the digital image analysis software ImageJ (ImageJ software, version 1.51, USA) was used for image processing, measurement, and analysis, as previously reported (10). Using the ‘area method’, the total area occupied by positively and negatively stained regions can be selected using ImageJ’s threshold tool. Positive IHC and Masson indices of the images were calculated.

### Statistical analysis

Statistical analyses were performed using MedCalc software, version 19.0 (MedCalc Software, Mariakerke, Belgium) and IBM SPSS Statistics for Windows, version 20.0 (IBM Corp, Armonk, NY, USA). *p*< 0.05 was the threshold for a statistically significant difference. Interobserver consistency was calculated using the intraclass correlation coefficient (ICC) with a two-way random effects model. Student’s t-test and one-way analysis of variance (ANOVA) were used to test the differences between T1 mapping parameters and differentiation types, T and N stages, and venous and neural invasion. Data were normally distributed using the Kolmogorov-Smirnov test. Pearson’s correlation analysis tested the correlation between T1 mapping, CDX-2, Ki-67 index, and collagen expression.

## Results

### Interobserver variability

**Figure 2:**
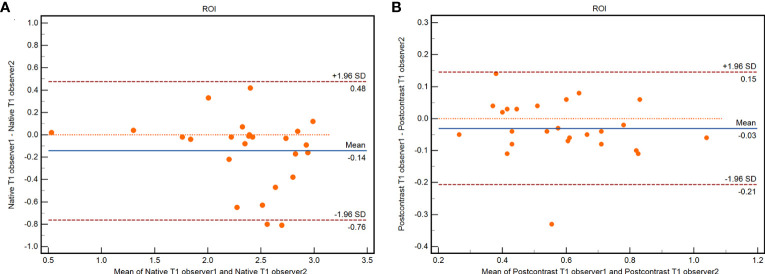
Bland-Altman plots of interobserver agreement for ROIs in native T1 value **(A)** and postcontrast T1 value **(B)**.

Excellent interobserver reproducibility was observed during the measurement of native and postcontrast T1 values derived from the T1 mapping based on different ROIs from the two observers (native T1 value ICC = 0.945, 95% confidence interval (CI): 0.877–0.976; postcontrast ICC = 0.942, 95% CI: 0.871–0.974). Graphic illustrations of interobserver variability using Bland–Altman plots are shown in **Figure 2**.

The mean native and postcontrast T1 values and ECV were 2.48 ± 0.45 ×10^-3^ msec (range, 1.30×10^-3^ msec - 3.09×10^-3^ msec), 0.58 ± 0.19 ×10^-3^ msec (range, 0.27×10^-3^ msec - 1.04×10^-3^ msec), and 53.17 ± 8.8% (range, 31.78%-70.85%), respectively.

### Pathological findings

The mean ratios of CDX-2, Ki-67, and collagen expression were 36.1% ± 11.5% (range, 16.6% - 70.1%), 27.5% ± 9.9% (range, 13.5% - 51.7%), and 18.4% ± 5.9% (range, 6.2% - 27.5%), respectively.

Significant differences were observed in CDX-2 and Ki-67 between low grade and medium-high grade (48.0 ± 13.4% and 32.2 ± 7.7%, t=3.631, *p*=0.001; 37.1 ± 9.4% and 24.3 ± 8.0%, t=3.264, *p*=0.004) on the WHO grading scale.

A strong positive correlation was found between CDX-2 and Ki-67 levels (r=0.816, *p*=0.000) and a negative correlation between CDX-2 and collagen fibers (r=-0.413, *p*=0.045).

### MRI findings

Postcontrast T1 values showed significant differences between venous invasion t=2.497, *p*=0.021) and neural invasion (t=2.254, *p*=0.034). However, no significant differences existed between native T1 values and ECV, differentiation type, T stage, and N stage. The results are presented in **Table 2**.

**Table 2:** Correlation between T1 mapping parameters and patients characteristics.

		Native T1 value (×10^-3^ msec)	Postcontrast T1 value (×10^-3^ msec)	ECV (%)
Histologic Grades				
Low Grade	6	2.35 ± 0.45	0.55 ± 0.17	49.15 ± 10.03
Medium Grade	12	2.50 ± 0.47	0.62 ± 0.21	55.61 ± 7.61
High Grade	6	2.56 ± 0.47	0.55 ± 0.19	52.33 ± 9.70
F		0.325	0.429	1.126
*P*		0.726	0.656	0.343
T stages				
T2	3	2.27 ± 0.90	0.58 ± 0.15	50.55 ± 3.03
T3a	11	2.39 ± 0.40	0.54 ± 0.17	52.08 ± 11.20
T3b	4	2.70 ± 0.15	0.68 ± 0.33	55.02 ± 10.02
T3c	6	2.59 ± 0.43	0.60 ± 0.13	55.26 ± 5.19
F		0.769	0.583	0.286
*P*		0.525	0.633	0.835
N stages				
N0	12	2.44 ± 0.45	0.61 ± 0.21	55.01 ± 5.98
N1	6	2.60 ± 0.46	0.56 ± 0.16	52.39 ± 13.58
N2	6	2.43 ± 0.52	0.55 ± 0.17	50.29 ± 8.64
F		0.270	0.214	0.584
*P*		0.766	0.809	0.567
Venous invasion				
Negative	15	2.54 ± 0.48	0.65 ± 0.18	52.99 ± 9.66
Positive	9	2.37 ± 0.40	0.47 ± 0.15	53.48 ± 7.69
*t*		0.890	2.497	-0.127
*P*		0.383	0.021	0.900
Nerve invasion				
Negative	16	2.48 ± 0.51	0.64 ± 0.19	51.92 ± 10.21
Positive	8	2.47 ± 0.35	0.47 ± 0.14	55.68 ± 4.52
*t*		0.093	2.254	-0.984
*P*		0.927	0.034	0.336

**Figure 3:**
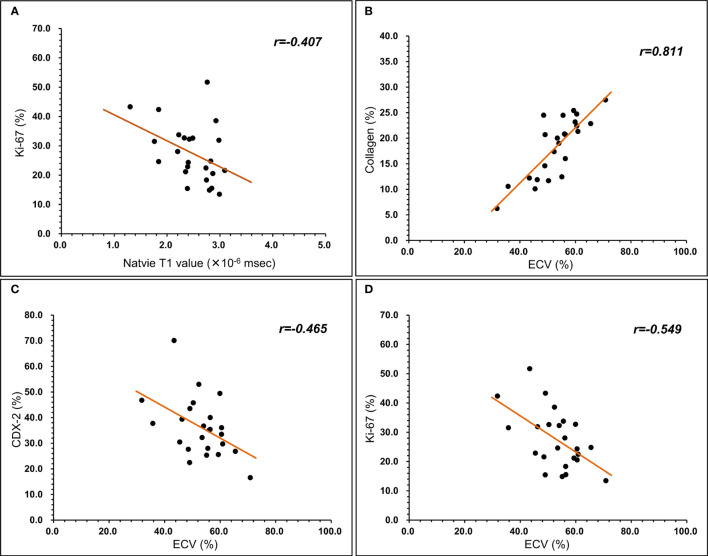
Relationship between T1 mapping parameters and histopathological component. **(A)** native T1 value was negatively correlated with Ki-67 (r=-0.407, P<0.05). **(B)** ECV was positively correlated with collagen (r=0.811, P<0.05). **(C)** ECV was negatively correlated with CDX-2 (r=-0.465, P<0.05). **(D)** ECV was negatively correlated with Ki-67 (r=-0.549, P<0.05).

**Figure 4:**
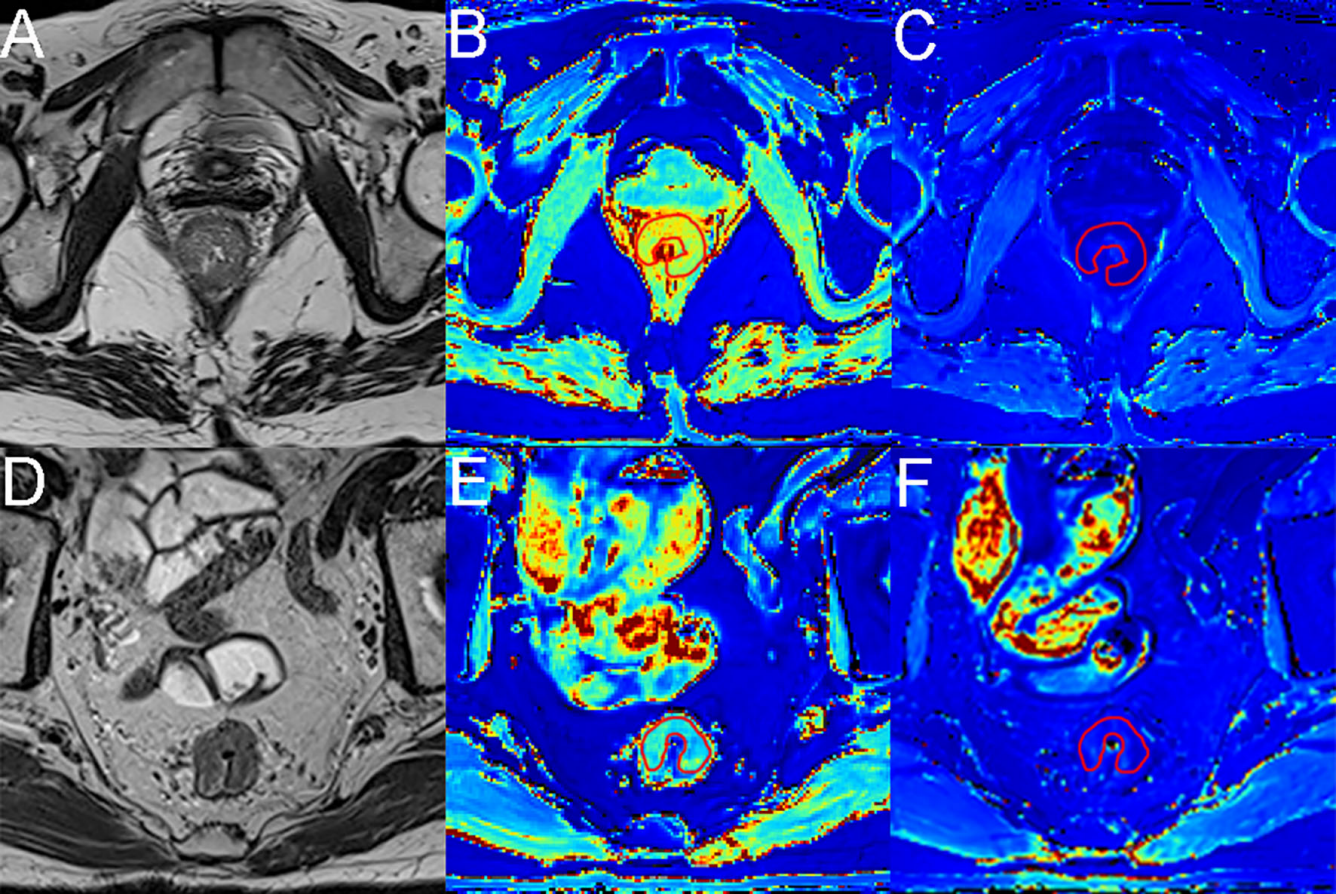
MR images from a 56-year-old female patient and a 69-year-old male patient with rectal cancer. **(A, D)** Axial T2-weighted images show hypointense masses in the rectum. **(B, E)** Axial native T1 mapping images, with native T1 values of 2.99× 10^-3^ msec and 1.76× 10^-3^ msec for the lesions. **(C, F)** Axial postcontrast T1 mapping images, with native T1 values of 0.38× 10^-3^ msec and 0.61× 10^-3^ msec for the lesions. The red lines represent the ROI outlined.

**Figure 5:**
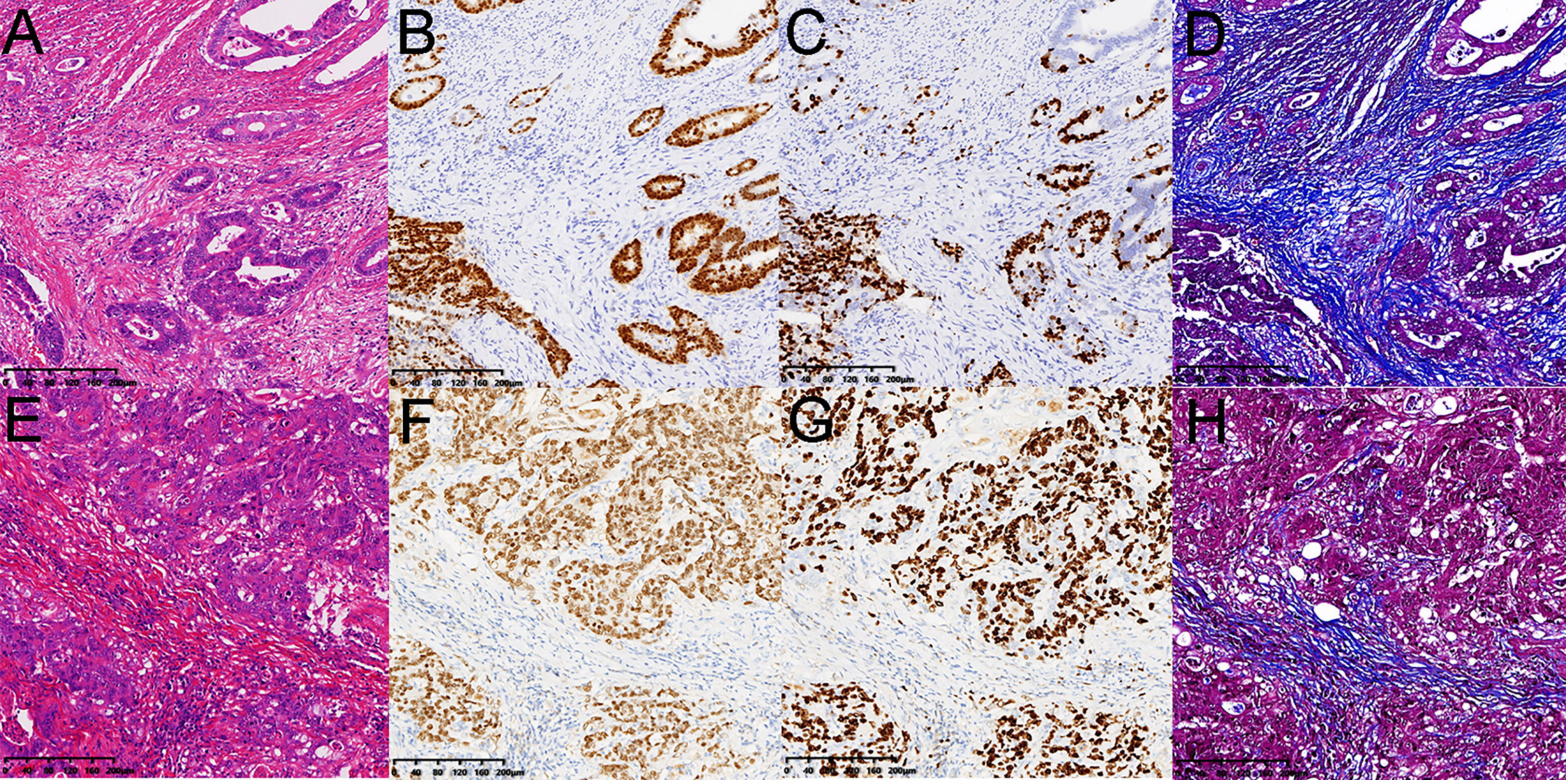
Pathology images from a 56-year-old female patient and a 69-year-old male patient with rectal cancer as the same as **Figure 4**. **(A, E)** hematoxylin and eosin-stained sections. **(B, F)** the CDX-2 ratios were 16.89% and 37.72%. **(C, G)** the Ki-67 ratios were 13.48% and 31.52%. **(D, H)** the collagen fibers ratios were 27.51% and 10.57%. The ECV fractions were 70.85% and 35.74%.

Pearson’s correlation analysis showed a significant negative correlation between native T1 values and Ki-67 (r=-0.407, *p*=0.049; **Figure 3A**). A significant positive correlation was found between ECV and collagen (r=0.811, *p*=.000; **Figure 3B**) and a significant negative correlation between ECV and CDX-2 (r=-0.465, *p*=0.022; **Figure 3C**) and Ki-67 (r=-0.549, *p*=0.005; **Figure 3D**). As shown in **Figures 4**, **5**, a 56-year-old patient with lower ratios of CDX-2 and Ki-67 and a higher collagen fiber ratio showed higher of ECV, compared with a 69-year-old patient with higher percentages of CDX-2 and Ki-67 and a lower ratio of collagen fiber.

## Discussion

Our study prospectively evaluated the correlation between quantitative T1 mapping parameters and tumor tissue composition for patients with rectal cancer. Our first main finding was that postcontrast T1 values showed lower T1 values in venous invasion and neural invasion in rectal cancer. Secondly, ECV was positively correlated with collagen and negatively correlated with CDX2 and Ki-67, and native T1 was negatively correlated with Ki-67. Therefore, our study suggests that the T1 value and ECV derived from T1 mapping can potentially evaluate rectal cancer invasion and quantify the tumor components of rectal cancer.

T1 mapping refers to the pixel representation of absolute T1 relaxation time on the map. Values extracted from T1 mapping have the advantages of excellent spatial resolution, repeatability, reproducibility, and accuracy. Native T1 values reflect a composite signal of cells (mainly cancer cells) and extracellular compartments. Elevations in native T1 can occur with edema, infiltrative processes, or fibrosis. Karamitsos et al. found that subjects with cardiac amyloidosis had significantly higher native T1 than those with aortic stenosis (11). Luetkens et al. found that native T1 values increased with increasing severity of liver fibrosis (12). However, we did not find a correlation between the native T1 values and collagen fibers in the extracellular interstitium.

In contrast to myocardial amyloidosis and liver fibrosis, the extracellular space in rectal cancer may have complex components, such as cystic degeneration and necrosis. This study found that native T1 values showed a significant negative correlation with Ki-67 expression levels. Ki-67 is a protein in the growing nucleus associated with cell proliferation (13). However, unlike the ECV, native T1 is influenced by intracellular and interstitial factors. Therefore, our study indicates that the native T1 value extracted via T1 mapping is mainly related to the number of proliferating rectal cancer cells. Another study found that the native T1 relaxation times of the low-grade rectal adenocarcinoma groups were significantly lower than those of the high-grade group (14). Our study also found that the mean native T1 values of rectal cancer in the low-grade group were lower than those in the medium-high-grade group. At the same time, we discovered that CDX-2 and Ki-67 levels in the low-grade group were significantly higher than those in medium-high grades. The increased native T1 values in high-grade rectal cancer patients may be due to the more epithelial cells and proliferative cells within the tumor (15). However, no statistically significant difference was found in our study. This may be related to the small sample size, and a larger sample size is needed for further conclusions.

After the gadolinium injection, extracellular contrast agents are distributed in the extracellular volume. Postcontrast T1 mapping has been used to differentiate recurrence and radio necrosis of brain metastases after gamma knife radiosurgery (3). Zach et al. showed that delayed contrast MRI used for calculating treatment response assessment maps could also differentiate tumor tissue from nontumor tissue in brain tumor patients (16). In this study, we found that the postcontrast T1 values of the positive group were significantly lower than those of the opposing group for venous invasion and nerve invasion. This may be because rectal cancer with venous and nerve invasion may have more neovascularization. Neovascularization is not mature and impaired, and the contrast agent cannot be effectively and quickly cleared. The compact structure of the tumor tissue may also lead to changes in pharmacokinetics. These factors lead to contrast agent accumulation and a decrease in the T1 value.

ECV extracted by T1 mapping has more significant advantages than native and postcontrast T1 values (17). ECV is not dependent on magnetic field strength and minimizes systematic errors in image acquisition. It was calculated by applying the formula above, including T1 values of tissue and blood before and after gadolinium contrast enhancement and red blood cell pressure volume, representing the intracellular portion of the blood cavity. This avoids confounding factors, such as blood T1 behavior, variable dose and clearance of contrast, and variation in hematocrit. ECV allows the direct measurement of the extracellular space (interstitial and extracellular matrix), dividing tissues into cellular (predominantly cancer cells) and extracellular interstitial compartments. These results were more consistent with the histological analysis. ECV increased due to extracellular matrix deposition and extracellular interstitial fibrosis. Luetkens et al. found that ECV increases with different severities of liver fibrosis (12). ECV has become a recognized alternative to diffuse fibrosis in the study of myocardial diseases (18). Cancer has more immature neovascularization and blood compartments than the myocardium, which may affect the ECV measurements.

In a recent study, the ECV fractions based on T1 mapping in the lymphovascular space invasion (LVSI) group were significantly higher than those in the non-LVSI group in patients with cervical cancer (5). Adams et al. found that MRI-derived ECV was substantially higher for higher-grade renal cell carcinoma (cRCC) than for lower-grade cRCC (19). Another study found a significant increase in ECV in thymic carcinomas compared with low-grade and high-grade thymomas (20). However, this study had no significant differences between T1 values, ECV, and differentiation types. Our study found that the percentages of CDX-2 and Ki-67 in low-grade rectal cancer were significantly higher than those in the medium to high-grade group.

In contrast, Ki-67 has been used to assess tumor proliferation and is associated with poor prognosis in many other tumor types, including breast, lung, and prostate cancers (21–23). The relationship between Ki67 expression and the degree of tumor differentiation appears to be different in rectal cancer compared to other tumor studies. In a study of 1,800 colorectal cancer samples, high Ki-67 expression was found to be an independent prognostic factor consistent with our study (24). Duchrow et al. estimated that many non-cycling tumor cells express Ki67 in at least one-third of CRCs cases. These Ki-67-positive non-cycling tumor cells may be more stable than tumor cells that fail to achieve cell cycle arrest and thus may be more resistant to adjuvant therapy or the patient’s immune response (25). A retrospective study found that tumor ECV was significantly higher in the chemotherapy-responsive group than in the non-responsive group in pancreatic ductal adenocarcinoma (4). However, the cause of chemotherapeutic drug-induced ECV changes and the relationship between ECV and tumor components remain unclear.

Our study used immunohistochemical techniques to quantify intracellular components, including nuclei of epithelial cells and nuclei of cancer cells, and extracellular collagen fibers in the stroma to understand the relationship between ECV and intra-tumoral components. In rectal cancer, the rate of collagen content in the extracellular stroma decreases with a high percentage of cellular components. In rectal cancer, the rate of cellular components is negatively correlated with the rate of collagen content in the extracellular stroma. Our study found that epithelial CDX-2 positive cells and Ki-67 positive cancer cells were negatively correlated with ECV, while collagen fibers were significantly positively correlated with ECV. Our findings indicate the ECV derived from T1 mapping might be a robust marker for assessing cells and collagen fibers in the extracellular stroma in rectal cancer.

T1 mapping and ECV appear to be reliable methods for venous invasion, nerve invasion, and identification of tumor components in rectal cancer. Just as the native T1 map can be viewed as an intrinsic tissue contrast agent, ECV is a direct measure of the size of the extracellular space, reflecting the interstitial area. This technique separates tumor tissues into cellular and interstitial components. These technologies are promising for the early detection of vascular and nerve invasion in rectal cancer, assessment of the internal components of colorectal cancer, and providing tools for more personalized treatment and monitoring of the effects of chemoradiotherapy. Thus, T1 mapping and ECV may serve as biomarkers to identify rectal cancer’s invasion and tumor composition. However, studies with larger samples from more centers are needed before recommendations can be made for clinical decision-making.

However, the current study has some limitations. First, it was based on a small sample of data from a single center. Second, we selected the layer with the most profound tumor infiltration to draw ROIs and sample pathological tissues. However, these may be different. Future studies should focus on more accurate tumor ROIs and pathological sampling localization. Third, in animal models of cardiomyopathy, studies have shown no significant change in cardiac ECV obtained by T1 mapping at 3, 7, 10, 15, and 20 minutes after the injection of contrast (26). However, further studies are needed to reliably estimate the minimum scanning delay time for ECV in rectal cancer.

## Conclusions

In conclusion, ECV measurements based on T1 mapping may represent an *in vivo* biomarker for detecting cells and collagen fibers in rectal cancer. Therefore, ECV may become a valuable MRI imaging marker, which can monitor intratumoral components and evaluate the effects of radiotherapy and chemotherapy.

## Data availability statement

The original contributions presented in the study are included in the article/supplementary material. Further inquiries can be directed to the corresponding author.

## Ethics statement

The studies involving human participants were reviewed and approved by Institutional Review Board (IRB) of Shuguang Hospital affiliated with Shanghai University of Traditional Chinese Medicine (2019-750-105-01). The patients/participants provided their written informed consent to participate in this study. Written informed consent was obtained from the individual(s) for the publication of any potentially identifiable images or data included in this article.

## Author contributions

JY, WT, and ZG designed this study. JY, QW, HW, JW and KL collected data. JY, SZ, JW and ML analyzed the data. JY wrote the manuscript text. All authors contributed to the article and approved the submitted version.
